# Efficient SNP Discovery by Combining Microarray and Lab-on-a-Chip Data for Animal Breeding and Selection

**DOI:** 10.3390/microarrays4040570

**Published:** 2015-11-16

**Authors:** Chao-Wei Huang, Yu-Tsung Lin, Shih-Torng Ding, Ling-Ling Lo, Pei-Hwa Wang, En-Chung Lin, Fang-Wei Liu, Yen-Wen Lu

**Affiliations:** 1 Department of Animal Science, National Taiwan University, Taipei 10617, Taiwan; E-Mails: d98626004@ntu.edu.tw (C.-W.H.); sding@ntu.edu.tw (S.-T.D.); demonwang@ntu.edu.tw (P.-H.W.); eclin01@ntu.edu.tw (E.-C.L.); 2 Department of Bio-Industrial Mechatronics Engineering, National Taiwan University, Taipei 10617, Taiwan; E-Mails: pqf292@gmail.com (Y.-T.L.); b97504092@ntu.edu.tw (F.-W.L.); 3 Department of Animal Science, Chinese Culture University, Taipei 11114, Taiwan; E-Mail: loll@faculty.pccu.edu.tw

**Keywords:** economic traits, swine, chicken, marker-assisted selection (MAS)

## Abstract

The genetic markers associated with economic traits have been widely explored for animal breeding. Among these markers, single-nucleotide polymorphism (SNPs) are gradually becoming a prevalent and effective evaluation tool. Since SNPs only focus on the genetic sequences of interest, it thereby reduces the evaluation time and cost. Compared to traditional approaches, SNP genotyping techniques incorporate informative genetic background, improve the breeding prediction accuracy and acquiesce breeding quality on the farm. This article therefore reviews the typical procedures of animal breeding using SNPs and the current status of related techniques. The associated SNP information and genotyping techniques, including microarray and Lab-on-a-Chip based platforms, along with their potential are highlighted. Examples in pig and poultry with different SNP loci linked to high economic trait values are given. The recommendations for utilizing SNP genotyping in nimal breeding are summarized.

## 1. Introduction

In recent years, breeding programs for farm animals have dramatically evolved from visual and phenotypic evaluations with subjective judgments to quantitative selections with genetic technology. While the breeding selection based on phenotypic evaluation can be slow, difficult and often not completely accurate for quantitative traits (also “polygenic” or “complex” traits), marker-assisted selection (MAS) techniques, which allow breeders to select farm animals with high breeding values early and effectively, have gained high popularity. It is a selection process that focuses on the traits of interest based on the linked genetic markers [1]. When it is coupled with quantitative trait loci (QTL) analysis, MAS assists breeders to locate genetic loci associated with quantitative traits to select the individuals with desirable combinations of the genes [2,3,4].

MAS typically utilizes three types of genetic markers: (i) direct marker, for causative mutations, (ii) linkage disequilibrium (LD) maker, for population-wide linkage disequilibrium with the QTL, and (iii) linkage equilibrium maker (LE), for population-wide equilibrium with the QTL within pedigree [5]. The MAS aims to maximize the rate of improvement in quantitative characters under different schemes of MAS information in molecular genetic polymorphism with data on phenotypic variations among individuals [1]. Genetic markers used in MAS include random amplified polymorphic DNA (RAPD), single sequence repeat (SSR), amplified fragment length polymorphism (AFLP), and single nucleotide polymorphism (SNP). As they show their applicability in animal breeding to select qualitative and quantitative traits [6,7,8,9,10], SNP markers have increasingly attracted great attention, particularly for germplasm diversity evaluation due to the highly qualitative nature of data (QND) and the highly effective marker index (EMI) [10,11,12].

Moreover, SNP markers are abundant in the genome, genetically stable, capable of effectively distinguishing different alleles, and amenable for automated high-throughput analysis [9,13]. The combination of SNP genotyping techniques and large-scale association study can further identify the relationships between genes and SNP markers of single-gene traits, QTL or genomic regions affecting quantitative traits [3,4]. Therefore, effective SNP genotyping with high accuracy is important to the success of highly efficient breeding selection.

A variety of SNP genotyping techniques have been developed to improve their detection accuracy, time, and cost [14]. These genotyping procedures typically involve the amplification of allele-specific products for the SNP of interest. This is followed by the detection techniques, such as enzymatic ligation [15,16], enzymatic cleavage [17,18], primer extension [19,20], split DNA enzymes G-quadruplex [21,22], sequencing [23,24], and mass spectroscopy [25]. These detection techniques utilize enzymes, molecular beacon, or fluorescent dyes to label the DNA probes, thereby leading to the requirement of high reagent cost or complex procedures.

Utilizing SNP genotyping techniques to select the species of farm animals with high economic trait values, including reproduction, growth rate, milking yield, and egg production, have become increasingly popular [26]. Recently, great efforts have been devoted to improving the SNP detection efficiency, reducing reagent consumption, minimizing sample volume and increasing sensitivity based on microfabrication techniques [27,28,29]. The application of genotyping procedures in farm animals should be simple and affordable, while they can simultaneously generate a vast amount of genotyping data [13,30,31]. A miniaturized version of the genotyping procedures in farm animals can further reduce the associated cost and make point-of-care gene detection/diagnosis possible [32]. Particularly, in recent years, miniaturized devices, which can integrate a series of laboratory functions on a single tiny chip (or so-called Lab-on-a-Chip), have many advantages over their analogues at the macroscale, including portability, reduced sample consumption, rapid reaction times, and high throughput [33,34,35,36,37,38]. For instance, SNP discrimination of swine in reproduction traits has been demonstrated [39]. Hence, the potential of Lab-on-a-Chip technology combined with SNP genotyping provides a number of advantages, which include decreasing the amount of reagents for the experiments and shortening the time for genetic selection, allowing breeders to conduct SNP detection on-site and to *ad-hoc* select candidates for high value breeding.

The purpose of this article is to discuss the procedures of SNP discovery and genotyping techniques in regard to selective animal breeding. The associated SNP information and genotyping techniques, including microarray and Lab-on-a-Chip based platforms, are highlighted. The recommendations of utilizing SNP genotyping for animal breeding are summarized.

## 2. Discovery of Novel SNP (Single Nucleotide Polymorphism) for Animal Breeding

**Figure 1:**
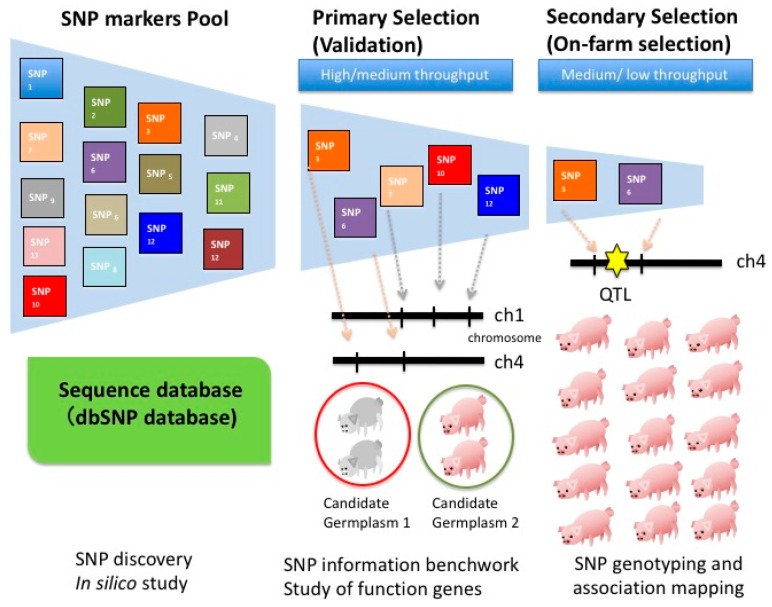
Procedures to identify SNP (single nucleotide polymorphism) markers with high economic traits of interest for selective breeding: (1) Breeders use *in silico* study to obtain SNP markers (shown in the blocks in different colors) from a SNP markers pool or public database (e.g., Single Nucleotide Polymorphism Database, or dbSNP); (2) Breeders conduct the primary selection or pilot study to validate SNP markers from the whole genome or putative functional genes based on *in silico* study. The primarily selected SNP markers are to be estimated as the polymorphism between two distinct small germplasm pools. Usually, DNA collected from 30–35 individuals of the same line is mixed as a representative germplasm with the other representative germplasm achieved from a distinct pool of 30–35 individuals. Only polymorphic SNP markers are chosen for the next step; (3) The secondary selection is achieved by using polymorphic SNP markers and a huge population to conduct the association analysis. The highly associated SNP markers with the quantitative trait loci (QTL) (red star) can be used as a potential genetic marker on marker-assisted selection (MAS).

SNP markers have been utilized by breeders to seek for high breeding values of economic traits [40]. They are used to predict the potential genes associated with economic traits, the linkage disequilibrium (LD) extent between markers at the genome level, as well as the livestock genetic diversity [41,42,43,44,45]. For example, researchers have used the SNP panel to estimate the LD levels of the pig breeds in Finland and United States [41]. The results show the SNP markers not only can serve as a powerful tool in MAS in breeds, but also reveal the phylogenetic relationships [41,46,47]. Further, more than 4000 QTL for production traits, with their associated SNP and SSR markers, are reported in chicken [48]. However, among a variety of available SNP markers and the sequence information of farm animals, only a few SNP markers contribute to the genetic variation for economic traits. The capability to effectively select critical SNP markers contributing to high breeding values is extremely relevant for efficient animal breeding schemes. Figure 1 illustrates a typical SNP selection procedure for animal breeding in three steps: (1) SNP discovery from a SNP marker pool, (2) SNP primary selection to validate the SNP in population and (3) secondary selection for routine SNP detection in nucleus farms.

In the first step of SNP discovery, SNP marker information is obtained through sequencing techniques, targeting local lesions in genomes (TILLING) analysis or *in silico* study on sequenced species. A SNP marker pool, from public database or Single Nucleotide Polymorphism Database (dbSNP), to represent informative SNP markers, is found at a given representative population. Based on the phenotypic differences and genetic background from the population with the traits of interest, the candidate SNPs are selected. Next, the primary selection is conducted to identify the association of the SNP markers and breeding germplasms. Bead-based assay and microarray are popular tools during this selection step. Finally, in the secondary selection step, the SNP markers validated from the primary selection are used during the association analysis. Additional samples from farms are exploited to evaluate their SNP genotypes. Commercial tools of TaqMan^®^ and Invader^®^ assays are commonly employed.

## 3. Strategies for SNP Discovery

To establish the large numbers of SNPs, two strategies have been employed in a given population. The first strategy is sequence-independent, and it detects unknown sequence variants or those species whose whole genome sequence are not available [49]. This strategy include restriction enzyme-based [50,51], DNA conformational changes, and target induced local lesions in genomes (TILLING) [52]. The traditional sequencing techniques such as shotgun sequencing or Sanger’s method are labor-intensive, time-consuming, and with relatively high error rates. Once a species’ genome has been sequenced and assembled, the re-sequencing of other individual species allows users to discover sequence variations on a genome-wide scale [53]. This strategy can provide indirect evidence, with solid evidence of sequence information still necessary to determine the actual genetic code.

The second strategy is sequence-dependent, and it detects known sequence variants, in which whole genome sequence information is utilized to discover SNPs of interest. Commercial genotyping assays can simultaneously genotype up to 2 million SNP per DNA sample (e.g., Illumina SNP chips). Other genotyping platforms involving multiplex, loci-specific PCR or whole genome amplification followed by primer extension or allele-specific hybridization can distinguish between bi-allelic SNPs in different fluorescence colors.

To interpret the sequencing data and to accurately identify the SNPs of interest, bioinformatics algorithms for searching SNPs have been developed, including Tablet [54], Pyrobayes [55], SOAP [56], VarScan [57], MAQ [58], MagicViewer [59], Atlas-SNP2 [60]. Hence, SNP discovery can also result from DNA resequencing analysis using novel deep-sequencing strategies. Although the sequence-dependent strategy is powerful, it relies on available sequence information and assembly of the sequencing contigs with a high rate of accuracy. On the other hand, the traditional sequence-independent strategy is irreplaceable for the species with limited sequence information available. In addition, the overlapping sequencing peaks of heterozygous alleles prove challenging in distinguishing these from sequencing errors and ambiguities, which can be easily distinguished using traditional strategies. These traditional methods are more feasible in genetic association mapping and diversity analysis by means of SNPs.

## 4. SNP Genotyping Technology

A variety of SNP genotyping techniques have been developed and reviewed [13,14,61,62,63,64,65,66,67,68]. These methods comprise a single or multiple allele-discrimination principles, along with signal detection mechanisms. One key feature in these SNP genotyping techniques, apart from those based on direct hybridization, is the two-step separation, which involves the first step in generation of allele-specific molecular reaction products and the second step in separation and detection of the allele specific products for SNP identification. Based on the molecular reaction system, SNP detection methods can be divided into four categories: primer extension-based, sequencing-based, restriction enzyme-based, and hybridization-based systems.

In this section, SNP genotyping techniques will be briefly discussed according to three categories, based on the number of samples or SNP sites to be genotyped. “Large throughput” genotyping is defined as genotyping of thousands of individuals for hundreds/thousands of SNP sites, or even conducting genome-wide association studies (GWAS). Likewise, “medium throughput” genotyping is an intermediary option, which detects hundreds of samples for hundreds to thousands of SNP sites. “Small throughput” methods only detect one to tens of samples for tens of targets [61,69]. Depending on the number of SNP sites and sample size, researchers and breeders can design and choose appropriate genotyping techniques. For example, in a genetic-mapping study that requires surveying many SNP markers along the whole genome to identify the candidate alleles, large throughput methods should be implemented. In a phylogenetic analysis, which demands a large sample size but only a few SNP markers, small and medium throughput methods are to be utilized with a few SNP primers or probes as an economical approach.

Table 1 lists the common SNP genotyping methods based on their throughputs in three categories: large, medium and small throughput. These intend to give breeders a general guideline to implement SNP genotyping techniques in farm animal breeding.

**Table 1:** Summary of the SNP genotyping platforms.

Genotyping Method	Proudct Name	Detection Mechanism	Platform	Throughput	Error Rate	Price	Reference
**Large Throughput** (more than hundreds or thousands SNPs/reaction)							
OpenArray^®^ (Applied Biosystems. Foster City, CA, USA)	TaqMAN	Primer extension	Fluorescence	>1000 samples/SNPs	Low	$$$	[45]
Infinium^®^ II (Illumina, San Diego, CA, USA)	Illumina Infinium assay	Primer extension	Fluorescence	>1000 SNPs/sample	Medium to High	$$$$$	[70]
GoldenGate^®^ (Illumina, San Diego, CA, USA)	GoldenGate^®^	Hybridization	Fluorescence	>1000 SNPs/sample/reaction	Low	$$$$$	[71]
Genome Wide SNP Array (Affymetrix, Santa Clara, CA, USA)	Affymetrix^®^	Hybridization	Fluorescence	>40 K SNPs/sample/reaction	Low	$$$$$	[72]
RAD sequencing	Illumina	Sequencing	Capillary electrophoresis	>13 K SNPs/sample/reaction	Low	$$$	[73]
Pyrosequencing	Pyrosequencing™	Sequencing	Pyrophosphate	>96 samples	Low	$$$	[74]
**Medium Throughput** (hundreds to low thousands SNPs/reaction)							
TaqMan assay (Applied Biosystems. Foster City, CA, USA)	TaqMan	Primer extension	Fluorescence	Up to 384 samples/SNP	Low	$$	[45]
MassARRAY^®^ system (Agena Bioscience, San Diego, CA, USA)	iPLEX	Primer extension	Mass spectrometer	60 SNPs/sample/reaction	Low	$$$	[75]
SNPstream genotyping system (Beckman Coulter, Brea, CA, USA)	48-plex GenomeLab SNPstream	Primer extension	Fluorescence	48 SNPs/sample/reaction	N.A.	$$$	[76]
SNaPshot^®^ multiplex system (Applied Biosystems)	SNaPshot	Primer extension	Capillary electrophoresis	10 SNPs/sample/reaction	Low	$$$	[77]
PCR-APEX	Genorama^®^	Primer extension	Fluorescene	Up to 384 samples/SNP	Low	$$$	[78,79]
Luminex xMAP technology (Luminex, Austin, TX, USA )	Luminex100™	Ligation	Flow cytometer	Up to 100 samples/SNP	Medium to high	$$$	[80]
**Small Throughput** (less than hundred SNPs/reaction)							
Invader assay	Laboratory use	Endonuclease cleavage	Fluorescence	1 SNP/sample/reaction	Low	$	[18]
PCR-RFLP	Laboratory use	Restriction enzyme	Gel electrophoresis	1 SNP/sample/reaction	Low	$	[51]
DASH	Laboratory use	Hybridization	Fluorescence	1 SNP/sample/reaction	Low	$	[81]

### 4.1. Large Throughput Methods

Large throughput genotyping methods deal with a large-scale project, where hundreds to thousands SNP sites on a few individuals or a few SNP sites on many individuals are simultaneously queried. These genotyping methods provide a great deal of SNP genotyping data, thereby enabling conducting whole-genome association studies in a given population.

**Figure 2:**
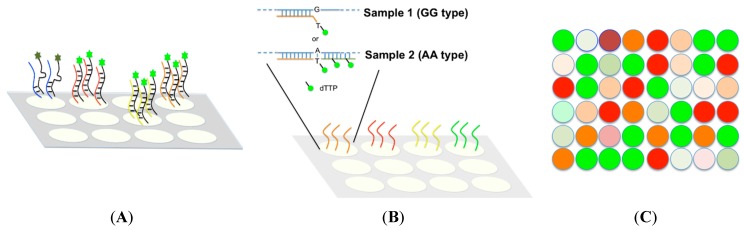
There are two types of microarray commonly used in multiplexing SNP analysis: allele-specific oligonucleotide (ASO) hybridization and allele-specific primer (ASP) extension. (**A**) ASO hybridization: The allele-specific oligonucleotide for every SNP is synthesized and separately immobilized onto the glass plate. Fluorescence labeled targets containing SNP sites are produced from a PCR reaction and plotted separately into each well to conduct the hybridization reaction. The mismatched base pair between target and oligonucleotide can decrease the binding strength with the fluorescence-labeled target removed after a stringent washing. A fluorescence signal is detected on a perfectly matched base pair; (**B**) Allele-specific primer (ASP) extension: The specific primer for SNP location is designed and separately immobilized onto a microarray. A different fluorescence labeled dNTP is individually used in an extension reaction. The extended fragment showing fluorescence signal can only be found when the 3′ end of primer pair is perfectly matched (AA type in this case) in contrast to the mismatched primer pair (GG type in this case); (**C**) The SNP genotype can be determined according to fluorescent intensity from the products/target DNA.

Microarray technology is an ideal method for large throughput analysis of multiple SNP sites. Two detection principles are widely used in microarray-based methods, which include allele-specific oligonucleotide (ASO) hybridization and allele-specific primer (ASP) extension, as shown in Figure 2. In Figure 2A, allele-specific oligonucleotides are separately immobilized onto the glass plate using the photolithography method. Fluorescent signal is detected upon the probe and target hybridized. The mismatched target is removed from the probe after a stringent washing procedure. An ASP extension-based microarray uses the primers pre-synthesized in an array to conduct PCR extension, as shown in Figure 2B, with extended product shown when 3′ end of primer perfectly bound to the sample target. By contrast, no extended product could be found when a mismatched base pair occurred at the 3′ end of the primer. Two types of microarray are used when determining multiple SNP genotypes by measuring fluorescence intensity or mass to charge ratio, as shown in Figure 2C.

Commercial tools derived from the concept of microarray have been developed; they include Infinium^®^ II (Illumina), GoldenGate^®^ (Illumina), Genome Wide SNP Array (Affymetrix, Santa Clara, CA, USA) and OpenArray^®^ (Applied Biosystems). Although these tools can provide massive information for SNP discrimination, two issues need attention: (i) the assay may have a high failure rate and (ii) it can only be conducted on model species because of the requirement of oligonucleotide probes or primers attached physically on the array [61,68]. In other words, lack of available oligonucleotide information may impede the use of large throughput microarray methods on the farm animals.

Despite the limitation in using microarray technologies, many advantages still exist, including large throughput data analysis and improving accuracy in accelerating animal breeding. For example, Jiang *et al.* [82] used BovineSNP50 BeadChip (Illumina) to conduct genome-wide association study (GWAS) for obtaining cattle milk production traits. They consequently screened 105 SNPs out of 54K SNPs, which are significantly associated genome-wise with one or multiple milk production on 2093 daughters from 14 paternal half-sib families.

Other large throughput SNP genotyping techniques include TaqMan^®^ OpenArray^®^, Restriction site-associated DNA sequencing (RAD sequencing) and Pyrosequencing. These techniques are flexible, high throughput and low cost. TaqMan^®^ OpenArray^®^ is the derivative of TaqMan product to discriminate multiple SNP genotypes [83]. It has been implemented in QTL mapping the pig meat quality gene, *PHKG1* with SNP markers. A total of 53 SNPs are first identified from Illumina PorcineSNP60 chip and further applied as an OpenArray^®^ for genotyping 140 pigs. A SNP site is found to affect *PHKG1* gene splicing during post-transcription and the mutant causes high glycogen content and low meat quality in skeletal muscle [83]. RAD sequencing and pyrosequencing are both developed for genotyping-by-sequencing (GBS) to discriminant sequence variant. RAD sequencing use fragmenting pooled samples’ genome for sequencing, which reduces the complexity across target genomes and delivers high resolution genomic data. The sequenced tags, RADSeqs, are used as the custom probes for massive parallel SNP genotyping [73]. A total of 50 QTL linked RADseqs are found to be highly associated with salmon resistance against Infectious Pancreatic Necrosis challenge [84].

### 4.2. Medium Throughput Methods

The second category is medium throughput SNP genotyping tools for a slightly smaller number of individual or SNP sites—hundreds of samples for hundreds to thousands of SNP sites. The commercial products in this category are iPLEX, SNPstream, SNaPshot, PCR-APEX (Arrayed primer extension), Luminex100™ and TaqMan assay. The first four methods are based on primer extension (iPLEX, SNPstream and SNaShot), while the latter two employ oligonucleotide ligation and exonuclease mechanism to achieve multiplexing in a single tube PCR. Similar concerns with large throughput methods remain—including limited applicability and high failure rate [61,68].

### 4.3. Small Throughput Methods

For small throughput projects, the technical platform for DNA molecular markers detection includes PCR-free genotyping methods, single-step homogenous method, homogeneous detection with fluorescence polarization, DNA chip/Array based assays, bead-based methods, mass spectrum based genotyping assays and dynamic allele-specific hybridization (DASH) [9,81]. Among the genetic markers used in these techniques, SNP markers are one of the preferred genotyping approaches due to their genetic stability and applicability to effectively distinguish heterozygote from homozygote alleles along with their co-dominances and amenable to high-throughput automated analysis [9,13].

PCR-RFLP is another technique for small throughput projects in which individual SNPs are differentiated by analyzing the patterns derived from cleavage of their amplified DNA. If a sample with different nucleotides in the same SNP site differs in the distance between sties of cleavage of a particular restriction endonuclease, then the length of the fragments produced differ when the DNA is digested with a restriction enzyme.

## 5. Miniaturizing Platforms of SNP Genotyping

### 5.1. Lab-on-a-Chip Platform

**Figure 3:**
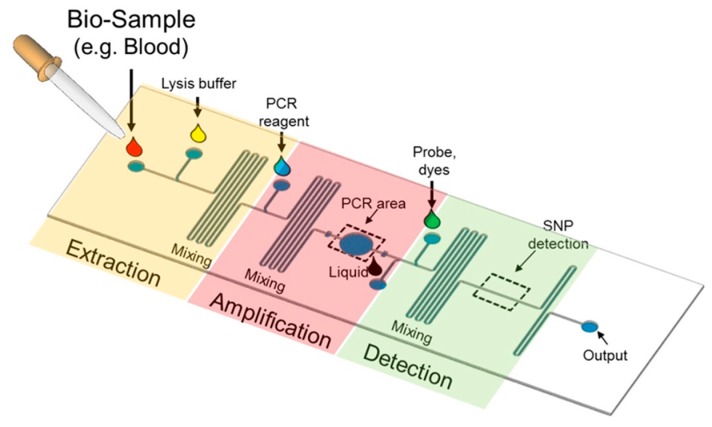
A typical Lab-on-a-Chip for SNP detection. Biological samples, which contain DNA, are injected into inlet into DNA extraction region. Blood cells are lysed. The crude DNA is produced and flows into the PCR area for amplification. PCR reagents including primer, dNTP and DNA polymerase are input into the channel to mix with DNA. The PCR product is then detected for SNP discrimination in the detection region.

Recently, miniaturized devices have brought many advantages over their analogues at the macroscale, including portability, reduced sample consumption, rapid reaction times, and high throughput [33,34,35,36,37]. In particular, Lab-on-a-Chip (LOC) involves the miniaturization of a series of sample preparation, genome amplification, and SNP detection onto a single chip, thereby simplifying the whole SNP genotyping process, as shown in Figure 3. It has been broadly used in biochemical fields, including genomics, proteomics, drug discovery, and infectious disease diagnostics. Such a miniaturization platform shows many advantages, including high surface-to-volume ratio, low reagent volumes, low background noise, and low reaction time.

Modern livestock production requires powerful technology for a fast, reliable and effective breeding protocol. For example, superior sows are selected based on their genetic background for high-yield traits. Once the piglets or poultry are selected at early stage, the breeders could save more money and increase the economic values. Lab-on-a-Chip technology, which implements genetic biotechnology onto a monolithic platform, enables the automation of the complete laboratory procedures of the genetic analysis protocol [48,85]. Use of this technology can simplify the analysis protocol, expedite the assay time, and reduce the risk of sample contamination.

For example, a Lab-on-a-Chip genotyping system, which integrated the functions of PCR and FRET-based melting curve analysis onto a microchip was developed to genotype SNPs—CYP3A4*15B allele and CYP3A5*3 allele [32]. Saliva samples from participants were directly used without any purification process, showing the potential of point-of-care SNP genotyping. A few more examples include the identification of plant pathogens [86], *Escherichia coli* (*E. coli*) and hepatitis B virus [87], human disease of non-syndromic sensorineural hearing loss (NSSNHL) [88], and many others.

### 5.2. Microfluidics for SNP Detection

The development of the Lab-on-a-Chip technology for SNP detection has been rapidly progressing. Various working principles had been utilized. A digital microfluidic system was developed for SNP detection [89]. A droplet containing magnetic beads and probes was actuated with addressable electrodes with the magnetic beads-probe used for SNP detection via the fluorescence signal due to oligonucleotide ligation [89]. Moreover, DNA in droplets or on beads within the channel had been developed for SNP detection. Recently, a hydrogel array for SNP detection was proposed with different probes separately incorporated in multiple channels to achieve multiplexing SNP detection [28]. This technique provided users with an opportunity for a large throughput analysis using a microfluidic-based system. An improved microarray device, made of a polyacrylamide gel-based microarray, was shown to overcome the drawbacks of the signal-to-noise ratio and diffusion-limited kinetics between the reaction liquids in which PCR or hybridization is conducted [90,91].

**Figure 4:**
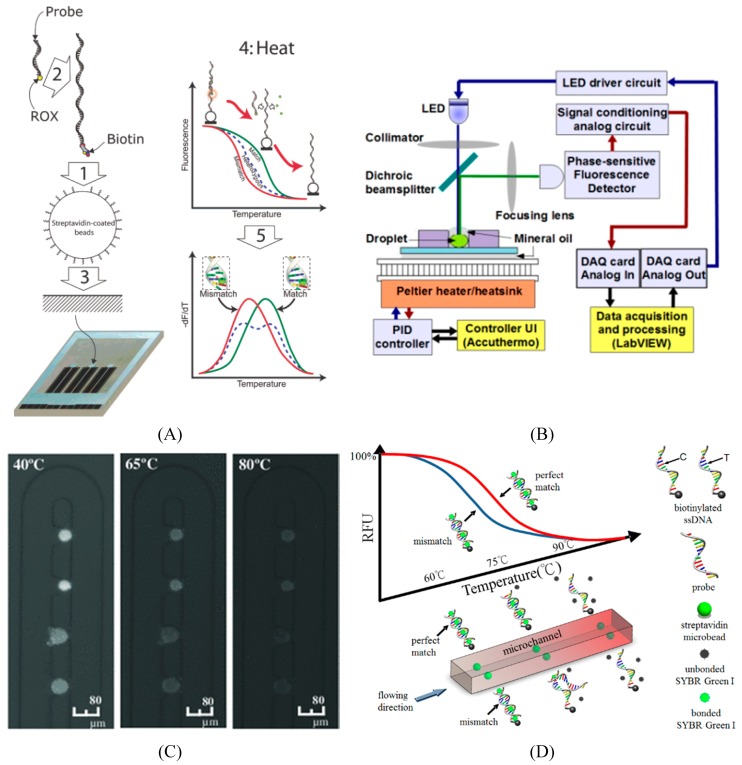

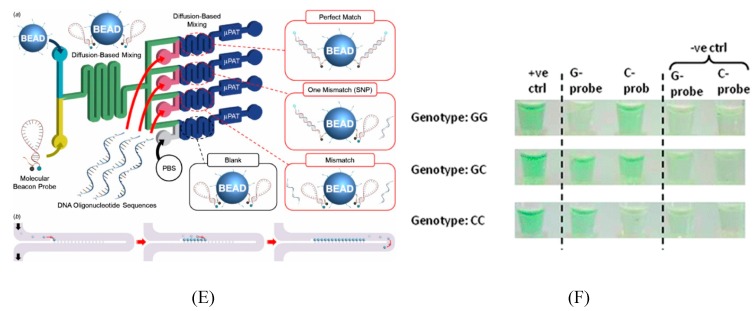
Examples of bead-based microfluidics as a Lab-on-a-Chip device for SNP detection. (**A**) Bead-based dynamic allele-specific hybridization (DASH) for SNP genotyping includes five steps: (1) the target DNA is isolated on beads, (2) an allele-specific probe is annealed, (3) the beads are monolayered by microcontact printing on the surface of the microheater while intercalating dye is added, (4) the chip is dynamically heated and (5) melting curve is obtained [92]; (**B**) The device used silica superparamagnetic beads to extract and carry sample DNA from mammalian. It performed sample preparation in droplet followed by real time PCR and employed melt curve apparatus for SNP detection [94]; **(C)** A bead-based SNP detection using melting temperature on a microchip. The target—probe-duplex-conjugated microbeads are hydrodynamically confined in microfluidic traps and heated. The corresponding fluorescent signals are recorded for melting curve analysis [39]; **(D)** Another version of bead-based SNP detection on a Lab-on-a-Chip, where melting analysis on microbeads is conducted in rapid temperature-gradient inside microchannels for possible genotyping in serial [95]; (**E**) A dynamic bead-based microarray for parallel SNP detection. Molecular beacon probes immobilized on microbeads, which are hydrodynamically arrayed on a micromachined chip, to quantitatively detect distinct DNA oligonucleotide sequences from the Hepatitis C viral (HCV) genome. Four types of samples, including perfect-match sample, one-mismatch sample (SNP), totally mismatch sample, and no sample control, were tested with microbeads [96]; (**F**) Visual SNP genotyping for SNP, across 3 different samples representing all three possible genotypes, GG, CC and GC. The intact peroxidase-like DNAzyme was used as a positive control. All PCR samples started withgenomic DNA and were tested in the presence of either β-G probesor β-C probes. Negative controls comprised split aptamers α and β-G probes or β-C probes in the absence of target DNA [22].

Due to its simplicity and low cost, melting curve analysis were widely applied for SNP genotyping in Lab-on-a-Chip. A rapid melting curve analysis system was demonstrated via immobilizing microbeads on the surface of a microheater chip with rapid temperature controller capabilities as shown in Figure 4A [92]. This method was based on random bead immobilization using a micro contact printing technique. A visual SNP genotyping system in Figure 4B was demonstrated via asymmetric PCR process and split DNA enzymes, G-quadruplex [21]. Only when both α-probe and β-probe were perfectly hybridized to the target single-stranded DNA, the G-quadruplex could be assembled. A hydrodynamic microbead array on a single chip combined with multiple bio-molecules detecting system was created [85]. Four types of samples, including (1) perfect-match sample, (2) one-mismatch sample (SNP), (3) totally mismatch sample and (4) no sample control, were injected to the microfluidic and mixed with microbeads, as shown in Figure 4C. A significant difference in fluorescence intensity was observed among four types of samples. While the system provided a multiplex detection microarray, it required fluorescence-labeled molecular beacons. In addition, a droplet-based SNP genotyping system with silica superparamagnetic beads in aqueous droplets was developed [93,94]. The device, whose schematics was shown in Figure 4B, utilized silica superparamagnetic beads to extract and carry sample DNA from mammalian. It performed sample preparation in droplet followed by real time PCR and employed melt curve apparatus for SNP detection. Different versions of bead-based SNP genotyping, which employed microbeads as solid carrier to conduct DNA melting analysis, were demonstrated for high signal-to-noise ratios [39,95]. The SNP genotyping based on melting curves was facilitated in microfluidic platform either being heated in a confined space or passing through in a rapid temperature gradient inside microchannels for conducting conduct DNA melting analysis, as shown in Figure 4C,D [39,95]. Discrimination of ataxia telangiectasia mutated (*ATM*) gene from Landrace sow was successfully shown. Another bead-based method was shown in a micromachined chip combined with multiple bio-molecules detecting system [96]. Four types of samples, including (1) perfect-match sample, (2) one-mismatch sample (SNP), (3) totally mismatch sample and (4) no sample control, were injected to the microfluidic and mixed with microbeads, as shown in Figure 4E. Finally, a visual SNP genotyping system in Figure 4F was demonstrated via asymmetric PCR process and split DNA enzymes, G-quadruplex [22]. Only when both α-probe and β-probe were perfectly hybridized to the target single-stranded DNA, the G-quadruplex could be assembled as a simple SNP genotyping approach.

## 6. Application of Trait Selection in Farm Animals by Using SNPs

The utilization of SNP genotyping techniques in promoting QTL analysis among farm animals will permit breeders and farmers to predict traits and estimate breeding values. The traits of economic interest in general include growth, body composition, carcass, meat quality, reproduction, and disease resistance capability [3,13,97,98]. These traits are mostly quantitative traits, and regulated by SNP. This section thus will review these popular traits, the associated genes, as well as their SNP markers. Two major farm animals with high economic values—swine and poultry—will exemplify our discussion.

### 6.1. SNP Discovery in Swine

In swine, (1) reproduction and (2) carcass and meat quality are traits with high economic values.

#### 6.1.1. Reproduction

The traits related to swine reproduction usually include litter size, litter number, number weaned, age at puberty, weaning to estrus interval, farrowing interval, and total number of pigs born (TNB) [99]. Among them, TNB is one of the most important reproductive traits. It includes (i) number born alive (NBA), (ii) number of stillborn piglets, and (iii) number of mummies [100]. The recent studies show that the development of early embryos, such as the period from morula, blastocyst for embryo implant, fetus development, and placental efficiency, is critical in determining the litter size of sow [101,102]. Landrace sows with specific SNPs located on the regulatory regions of *ATM* gene has been discovered by using GoldenGate^®^ in differentially expressed genes between morula and blastocyst [103]. These genes play an important role in TNB, NBA, and the average birth weight of piglets due to their differential expressions between the morula and blastocyst stages. Recently, the SNP associated to *ATM* genes had been detected by using Lab-on-a-Chip platforms [39,95].

Another important trait for reproduction is litter size, which is highly influenced by ovarian follicular growth. Two estrogens receptors (ESR) (e.g., *ESR1* and *ESR2*) are found to be involved in ovarian follicular growth [104,105]. The associated SNP has been genotyped using PCR-RFLP (Hsp92 II) method in two Iberian pig populations [105]. No significant association is found between litter size and *ESR2* polymorphism. Furthermore, the Polish sow with AA genotype is found to have the largest litter size compared to other genotypes of AB and BB [106]. Statistically insignificant trait difference in the results implies the utilization of a different SNP genotyping strategy with larger populations or more SNP markers may be necessary to identify the SNPs for the traits of interest. Up to now, all the SNPs identified in these two gene have failed to be validated in large populations or across breeds.

On the other hand, the polymorphism in Prolactin receptor (*PRLR*) gene, which controls luteal and follicular steroidogenesis of Large White sows (*n* = 301), has been tested by using the PCR-RFLP method. The results suggest that *PRLR/AluI* gene improves reproductive performance traits of sows [107]. The medium throughput genotyping techniques are adapted to validate multiple candidate SNPs and to decipher how *PRLR* and *ESR* play different roles in sow reproduction, without knowing the correlation with the traits of interests. In addition, a commercial PCR-APEX chip, which contains 45 SNPs, is used to determinate PSS-porcine stress syndrome (a single-gene disease) in four QTL genes, including protein kinase adenosine monophosphate-activated γ3-subunit (*PRKAG3*), calpastatin (*CAST*), Melanocortin 4 receptor (*MC4R*) and *ESR*—these four genes are exploited for the marker-assisted selection. The SNPs’ genotyping technique used in this selection procedure is PCR-APEX (Arrayed primer extension) technique—a medium throughput method [78,79].

Besides, there have been significant interest lately in several populations and in different approaches for leptin receptor (*LEPR*), *MC4R*, Phosphatidylinositol 3-kinase catalytic subunit type 3 (*PIK3C3*) and *vertnin* (*VRTN*) genes analyzed [98,99,100,101,102,103,104,105,106,107,108,109,110]. Associations of SNPs in inter-alpha-trypsin inhibitor heavy chains (*ITIH*) genes as well as retinol binding protein 4 (*RBP4*) and follicle stimulating hormone (*FSHB*) genes with porcine reproductive traits have been reported [111,112].

#### 6.1.2. Meat Quality Traits

Meat quality is important in swine-breeding programs. The related traits, including muscle growth, tenderness, color and oxidative stability, are mostly quantitative [12]. They are affected by many loci that have a wide degree of effects on phenotypic variations. For instance, muscle growth is found to be regulated by *MSTN* gene [4,45,113]. To estimate the LD, 351 animals in 117 sire/dam/offspring trios across four breeds of pigs (Duroc, Hampshire, Landrance, Yorkshire) using Illumina PorcineSNP60 BeadChip were used. Two SNP markers (g.435 and g.447) have been reported to associate with controlling myostatin expression in pigs. These two SNPs, located in the promoter region of *MSTN* gene, are vital functional genetic markers [45]. Insulin-like-growth factor 2 (*IGF2*) gene, located in chromosome 2 of pig, has been found to positively control skeletal muscle growth, which is discovered by using RFLP. An SNP (G>A) of *IGF2* intron 3 (g.3072) disturbs a repressor zinc finger BED-type containing 6 (*ZBED6*) binding onto *IGF2* and causes a three-fold overexpression of postnatal skeletal muscle *IGF2* mRNA. While this mutant leads to the increase of carcass lean yields, it reduces backfat deposition [114,115]. Furthermore, *IGF2* plays an important role in the pig industry due to the 15%~30% and 10%~20% of phenotypic variation in muscle mass and backfat thickness, respectively [116].

SNP marker of pituitary-specific transcription factor (*PIT1*) has been reported to be associated with growth and carcass traits in pigs [117]. The ryanodine receptor (*RYR1*) mutant pigs induce a high rate of glycolysis, leading to a low pH value of meat after slaughtering. The low pH value of meat could produce pale, soft, and exudative muscles, which have an unacceptable meat appearance for customers. *RYR1* gene was reported to be associated with porcine stress syndrome (*PSS*) and functions at regulating calcium transport across skeleton muscle [118]. Muscle regulatory factor (*MRF*) gene family, which encoded a basic helix-loop helix protein and was responsible for myotubular transformation. Two genes, *Myf5* and *Myf6*, in MRF family were found to regulate the development of skeletal muscle fibers and postnatal growth hypertrophy, which affected meat quality [118,119,120]. 

### 6.2. Poultry

Poultry has become the leading meat consumed in the United States and many other countries. It has been bred for two purposes: egg laying and meat production [43]. Therefore, the reproduction trait (e.g., egg production, egg quality and egg shell) and the meat production traits (e.g., muscle growth, tenderness and water holding capacity (WHC)) are of considerable economic importance [121,122]. The first SNP genotyping array for chicken has been demonstrated by Affymetrix^®^ lately, showing its importance in research and practical applications [123].

#### 6.2.1. Reproduction

Genetic selection in superior chicken improves the breeding speed and accuracy. Several important traits have been addressed in Taiwan chicken breeding programs, which include improvement of egg production, egg quality, and eggshell. The traits of egg production, egg quality and eggshell are possibly controlled by multiple genes. For instance, low density lipoprotein receptor-related protein 8 (*LRP8*) gene has a 6.5 kb receptor transcript and is expressed in the brain and ovary. The *LRP8* gene plays a role in cholesterol supply for steroid biosynthesis, thereby enabling folliculogenesis [124].

In birds, melatonin is found in ovarian follicular fluid, suggesting the role of melatonin on ovarian functions [125,126,127]. Two high-affinity melatonin receptors from *MSTN* family, *MTNR1A* and *MTNR1B*, have been cloned in numerous species, but there is an additional receptor subtype, *MTNR1C*, have been identified only in amphibians and birds. Direct PCR-sequencing, PCR-SSCP and PCR-RFLP are used to genotype these genes [125,126].

Also, the reproduction trait of poultry can be related to the laying performance. Due to low heritability, the selection in laying performance imperatively requires the MAS strategy. Yu et al. has 139,013 SNPs obtained from 42,291,356 RADseq tags by using RAD sequencing method [127]. Five novel genes, including membrane associated guanylate kinase (*MAGI-1*), *KIAA1462*, Rho GTPase activating protein 21 (*ARHGAP21*), acyl-CoA synthetase family member 2 (*ACSF2*) and astrotactin 2 (*ASTN2*) are found to be promising candidate MAS markers for egg number in geese.

#### 6.2.2. Growth

The haplotype of the *PIT1* gene has shown a significant association with growth traits in chicken. *PIT1* affects the expression of growth hormone, thyroid-stimulating hormone chain, and prolactin. The *PIT1* SNP marker has been used to determine the genotypes of 10 chicken populations (*n* = 662), including six Chinese indigenous breeds, White Leghorn, paternal/maternal lines of brown egg layer and a paternal line of broiler by using the PCR-SSCP method. Two *PIT1* SNP haplotypes, AA and TT types, have a significant difference between their body weights at eight weeks, making the *PIT1* SNP marker a potential marker for MAS selection of early growth rates in chicken [128]. Similar results have also been obtained by using PCR-RFLP in the cross of White Recessive Rock (WRR) and Chinese Xinghua [129].

In addition, the melanocortin 4 receptor (*MC4R*) belongs to G protein–coupled receptors (GPCR) super family and is a transmembrane neuron receptor, which controls the appetite, body weight and energy metabolism. The polymorphism of *MC4R* in the population of crossing Broiler and Chinese Silky chicken has been genotyped for the carcass traits in body weight and growth by using the PCR-SSCP technique [130].

Finally, all these candidate genes with SNP markers discussed in this section for economic traits of swine and poultry are summarized in Table 2.

**Table 2:** The SNP loci related to economic traits.

Species	Traits	Gene ^1^	Chromosome Location	Putative Functions	Refs.
**Swine**	Reproduction	*ATM*	9	Morula development	[103]
	Reproduction	*ESR*	1	Effect of follicular growth and litter size	[104,105].
	Reproduction	*PRLR*	16	Control luteal and follicular steroidogenesis	[107]
	Meat Quality	*MSTN*	15	Negative regulator for muscle mass	[4,45,113]
**Swine**	Meat Quality	*IGF-2*	2	Growth-promoting peptidesStructurally homologous with insulin Producing uniformity of pork leanness	[114,115]
	Meat Quality	*RYR1*	6	Known as Halothane gene, Ryanodine receptor causing Ca^2+^ release	[118]
	Meat Quality	*Myf5*, *Myf6*	5	Transcription regulator of skeleton muscle development and increase of meat mass	[118,119,120]
**Poultry**	Reproduction	*LRP8*	1	Cholesterol supply for steroid biosynthesis, which enables folliculogenesis, melatonin in ovarian	[124]
	Reproduction	*MTNR family*	8	*MTNR* binds melatonin, affecting growth and reproduction	[125,126]
	Reproduction (geese)	(*MAGI-1*), *KIAA1462*, *ARHGAP21*, *ACSF2*, *ASTN2*	*MAGI-1*: 12 *KIAA1462*: 2 *ARHGAP21*: 2 *ACSF2*: 18 *ASTN2*: 17	*MAGI-1*: cell proliferation and apoptosis *KIAA1462*: Meiotic recombination *ARHGAP21*: cell-cell adhesion formation and cellular migration *ACSF2*: fatty acid synthesis *ASTN2*: cell adhesion	[127]
	Growth	*PIT1*	4	Secretion of growth hormone, prolactin and thyroid-stimulating hormone	[128,129]
	Growth	*MC4R*	18	Appetite, growth and weight gain	[130]

^1^ *ATM*: ataxia telangiectasia mutated protein, *ESR*: Estrogen receptor, *PRLR*: prolactin receptor, *MSTN*: myostatin, *IGF-2*: Insulin-like-growth factor 2, *CRC*: calcium release channel, *MYF6*: myogenic factor 6, *LRP8*: low density lipoprotein receptor-related protein 8, *MTNR*: melatonin receptors, *MAGI*-*1*: membrane associated guanylate kinase 1, *KIAA1462*: KIAA1462, *ARHGAP*: Rho-GTP activating protein, *ACSF2*: acyl-CoA synthetase family member 2, *ASTN2*: astrotactin 2, *PIT1*: pituitary specific transcription factor gene 1, *MC4R*: Melanocortin 4 receptor.

## 7. Prospective

As the traits described in the last section are generally lowly heritable, traditional breeding selections with phenotypic evaluations are insufficient. Quantitative MAS techniques with reliable markers constitute a feasible and effective approach. In practice, depending on the number of individuals and SNP sites, choosing appropriate SNP genotyping techniques for animal breeding will involve tradeoffs between reliability, sample preparation, reagent/sample expense, instrument depreciation and procedure complexity. For instance, large throughput SNP methods require a series of complicated steps and special instruments. They are high cost, labor intensive and time-consuming.

Moreover, even though the rapid progress of sequencing technology and the “$1000 genome” project may be soon possible and can significantly reduce the cost [131], it may still be an unlikely ask to solely conduct whole genome sequencing (WGS) for breeding in farm animals. Instead, a feasible approach will be to utilize the WGS of a small population of the individuals and to use a SNP chip for the genotyping of the majority of the individuals should be collectively adapted. In other words, WGS is adapted for high accuracy during the first and second stages of the breeding (e.g., SNP discovery and primary selection in Figure 1), and a SNP chip is exploited for lower costs in the third stage (e.g., secondary selection in Figure 1). For example, during the stage of the secondary selection, successfully promoting the use of genetic selection in swine and poultry requires elevated animal breeding efficiency while maintaining high accuracy with high long range LD. In addition, using 10% of the SNP markers from the original panel can still result in good accuracy for genotype imputation [47], suggesting small or medium throughput techniques will be sufficient for SNP genotyping of certain traits of interest in breeding. Although it is at early stages, the Lab-on-a-Chip technique has shown its potential for low density panels in SNP genotyping and can become a feasible tool with practical demonstrations and industrial commercialization. This approach is particularly useful for farm animals whereby individuals are not as valuable as cattle or swine. However, breeding at the top levels (e.g., great grandparent (GGP) or grandparent (GP) lines) should be profitable because the profits can come from a large population descending from the genotyped and breed parent animals.

## 8. Summary

SNP genotyping techniques have received considerable attention in selective breeding. We have discussed the procedures of SNP discovery and genotyping techniques for animal breeding. The associated SNP information and various genotyping techniques are presented. General findings may be summarized as follows:

1. Animal breeding by using phenotypic selection is insufficient.
2. Utilization of genetic markers for breeding has become mainstream in the livestock industry.
3. In particular, breeding using SNP markers is an effective and economical approach as it possesses the advantages of abundance and wide distribution in genome, and is easy to analyze.
4. SNP markers can be discovered by using sequence-dependent and sequence-independent methods. The former one is large throughput but expensive, while the latter one is small throughput but robust.
5. Appropriate SNP genotyping techniques for animal breeding will be adapted addressing concerns regarding throughput, reliability, associated expense, and procedure complexity.
6. A combination of whole-genome sequencing for a small population of individuals and a SNP chip for the genotyping of the majority of individuals will be a feasible and economical approach for animal breeding.
